# New country records of reptiles from Laos

**DOI:** 10.3897/BDJ.1.e1015

**Published:** 2013-12-10

**Authors:** Vinh Quang Luu, Truong Quang Nguyen, Thomas Calame, Tuoi Thi Hoang, Sisomphone Southichack, Michael Bonkowski, Thomas Ziegler

**Affiliations:** † Department of Wildlife, Faculty of Natural Resource and Environmental Management, Vietnam Forestry University, Xuan Mai, Chuong My, Hanoi, Vietnam; ‡ AG Zoologischer Garten Köln, Riehler Strasse 173, D-50735 Cologne, Germany; § Institute of Ecology and Biological Resources, Vietnam Academy of Science and Technology, 18 Hoang Quoc Viet, Hanoi, Vietnam; | Zoological Institute, University of Cologne, Zülpicher Strasse 47b, D-50674 Cologne, Germany; ¶ WWF Greater Mekong, House No. 39, Unit 05, Ban Saylom, Vientiane, Lao People's Democratic Republic; # Biodiversity Center, Faculty of Natural Resource and Environmental Management, Vietnam Forestry University, Xuan Mai, Chuong My, Hanoi, Vietnam; †† Hin Nam No National Protected Area, Boualapha District, Khammouane Province, Lao People's Democratic Republic

**Keywords:** Colubridae, Gekkonidae, distribution, taxonomy, Khammouane Province, Salavan Province

## Abstract

Four species of reptiles, of which one is represented by one of its subspecies, are recorded for the first time from Laos: *Cyrtodactylus phongnhakebangensis*, *Lycodon futsingensis*, and *Lycodon ruhstrati*, as *Lycodon ruhstrati abditus*, from limestone forests in Khammouane Province and *Cyrtodactylus pseudoquadrivirgatus* from hill evergreen forest in Salavan Province. These discoveries of lizards and snakes bring the total species number of reptiles to 189 in Laos.

## Introduction

The knowledge on the diversity of the reptile fauna of Laos has strikingly increased during the recent decades. Stuart (1999) provided the first checklist of 109 reptile species from Laos. Teynié et al. (2004) published an updated checklist of reptiles from southern Laos with a total of 89 recorgnized species. Four years later, Stuart and Heatwole (2008) recorded 13 additional species of colubrid and viperid snakes from the country. The species number of reptiles from Laos was 180 in 2010 (Teynié and David 2010). Since 2010, six new species of reptiles have been described from Laos comprising *Cyrtodactylus wayakonei* Nguyen, Kingsada, Rösler, Auer & Ziegler, 2010, *Cyrtodactylus lomyenensis* Ngo & Pauwels, 2010, *Cyrtodactylus teyniei* David, Nguyen, Schneider & Ziegler, 2011, *Cyrtodactylus pageli* Schneider, Nguyen, Schmitz, Kingsada, Auer & Ziegler, 2011, *Lycodon davidi* Vogel, Nguyen, Kingsada & Ziegler, 2012, and *Oligodon nagao* David, Nguyen, Nguyen, Jiang, Chen, Teynié & Ziegler, 2012 (Nguyen et al. 2010, Ngo and Pauwels 2010, David et al. 2011, Schneider et al. 2011, Vogel et al. 2012, David et al. 2012). In 2012 and 2013, additional field surveys were conducted in the hill evergreen forest within Xe Sap National Protected Area, Salavan Province, and limestone forests within Hin Nam No National Protected Area, Khammouane Province. Examination of voucher specimens from aforementioned sites revealed the existence of a number of reptile species that have not been known from Laos so far. We herein report four new records of reptiles from the country, comprising two species of Gekkonidae, and one species and one subspecies of Colubridae.

## Materials and methods

**Figure 1.**
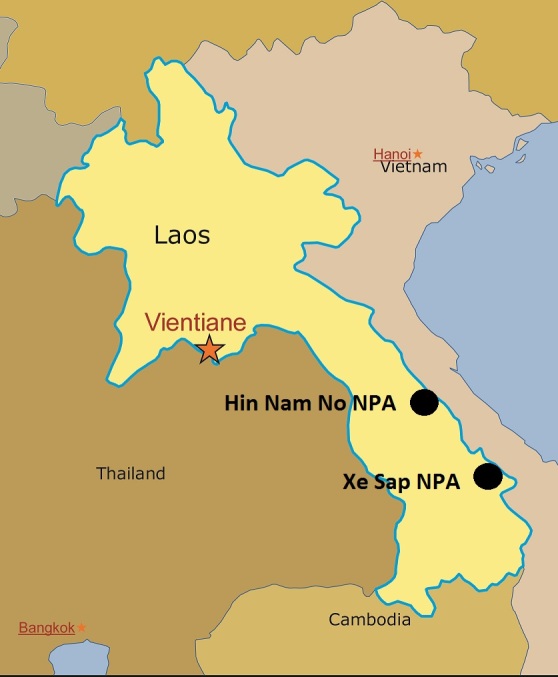
Map of survey sites in Khammouane and Salavan provinces, Laos.

Field surveys were conducted by T. Calame in Xe Sap National Protected Area (NPA), Salavan Province in May 2012 and by V. Q. Luu in Hin Nam No NPA, Khammouane Province from April to July 2013 (Figs 1, 2). Specimens were collected by hand or snake hook between 19:00-23:00. After taking photographs, specimens were anaesthetized, fixed in 80-85% ethanol and subsequently stored in 70% ethanol. Voucher specimens are deposited in the collections of the Institute of Ecology and Biological Resources (IEBR), Hanoi, Vietnam; the National University of Laos (NUOL), Vientiane, Laos; the Vietnam Forestry University (VFU), Hanoi, Vietnam; and the Zoologisches Forschungsmuseum Alexander Koenig (ZFMK), Bonn, Germany.

Measurements of specimens were taken with a digital caliper to the nearest 0.1 mm. Abbreviation are as follows: SVL (snout-vent length): from tip of snout to anterior margin of cloaca; TaL (Tail length): from posterior margin of cloaca to tip of tail; TL (total length): SVL+TaL. Terminology of morphological characters follows Rösler et al. (2008) for lizards and Vogel et al. (2009) for snakes. Bilateral scale counts were given as left/right.

## Taxon treatments

### Cyrtodactylus phongnhakebangensis

Ziegler, Rösler, Herrmann & Vu, 2002

#### Materials

- **Type status:** Other material. **Occurrence:** recordedBy: V. Q. Luu; individualCount: 9; sex: 4 males, 5 females; **Location:** country: Laos; stateProvince: Khammouane; verbatimLocality: Hin Nam No National Protected Area; verbatimElevation: 180-580 m; verbatimLatitude: 17º15'-17º40'N; verbatimLongitude: 105º43'-106º09'E; **Event:** eventDate: 2013-05-07/2013-06-30; **Record Level:** institutionCode: IEBR, VFU, NUOL, ZFMK

#### Description

(Fig. 3)

**Specimens examined (n = 9)**: Four adult males and five adult females, all collected by V. Q. Luu in Hin Nam No NPA, Khammouane Province: IEBR A.2013.89, adult male, 7 May 2013, from Hang Toi region, Noong Ma Commune (17°17.766’N, 106°08.803’E, elevation 580 m a.s.l.); VFU A.2013.1 and NUOL R-2013.2, adult males, 9 June 2013, from Vang Ma No Commune (17°30.778’N, 105°49.259’E, elevation 180 m a.s.l.); IEBR A.2013.90, adult male, 11 June 2013, from Ban Dou Commune (17°30.385’N, 105°49.160’E, elevation 183 m a.s.l.); ZFMK 95235, adult female, 8 May 2013, from Hang Toi region, Noong Ma Commune (17°17.763’N, 106°08.778’E, elevation 555 m a.s.l.); ZFMK 95236, adult female, 30 May 2013, from Noong Choong Region, Cha Lou Commune (17°20.248’N, 105°56.693’E, elevation 252 m a.s.l.); VFU A.2013.2-A.2013.3, adult females, 9 June 2013, from Vang Ma No Commune (17°30.778’N, 105°49.259’E, elevation 180 m a.s.l.); NUOL R-2013.3, adult female, 11 June 2013, from Ban Dou Commune (17°31.545’N, 105°49.086’E, elevation 197 m a.s.l.).

**Morphological characters:** SVL males 83.6-92.5 mm (mean ± SD 87.9 ± 4.9 mm), females 95-100.6 mm (mean ± SD 93.8 ± 5.0 mm); tail length (TaL) 101.6 mm in males, 108.3 mm in females; head depressed (HL/HW 1.6 in males, 1.5 in females), distinct from neck; snout longer than diameter of ocular (SE/OD 2 in males, 1.9 in females); snout scales small, homogeneous, granular, larger than those in frontal and parietal regions; rostral wider than high with a Y-shape in the middle; supranasals in contact; rostral bordered by first supralabial and nostril on each side; nares oval, surrounded by supranasal, rostral, first supralabial, and two enlarged postnasals; ear oval-shaped; mental triangular; postmental two, enlarged, in broad contact posteriorly; supralabials 9-12; infralabials 8-10; dorsal scales granular to flattened; dorsal tubercles triangular, conical, present on occiput, back and tail base, each surrounded by 8-9 granular scales, in 14-19 irregular longitudinal rows at midbody; ventral scales smooth, medial scales 2-3 times larger than dorsal scales, round, in 35-48 longitudinal rows at midbody; ventrolateral folds present; gular region with homogeneous smooth scales; precloacal groove absent; enlarged femoral scales present; femoral and precloacal pores 36-44 in males, pitted scales 0-28 in females; postcloacal tubercles 4-6; subcaudals enlarged; dorsal surface of fore and hind limbs with small tubercles; fingers and toes without distinct webbing; lamellae under fourth finger 16-21, under fourth toe 19-22. Coloration in preservative: Ground coloration of dorsal head and back greyish brown with dark spots; nuchal loop distinct, in U-shape, from posterior corner of eye through tympanum to the neck, dark brown, edged in white; body bands between limb insertions four to five, somewhat irregular, dark brown, edged in white; dorsal surface of fore and hind limbs with dark bars; tail brown dorsally with seven to eight light brown bands, edged in white; chin, throat, and belly cream; upper and lower lips with dark brown bars; tail ventrally grey with light dots (determination after Ziegler et al. 2002).

#### Ecology

Specimens were found between 19:00 and 22:00 on karst walls, ca. 0.5-3 m above the ground, near cave entrances in limestone forests, at elevations from 180 to 580 m a.s.l.

#### Distribution

*Cyrtodactylus phongnhakebangensis* has been known from Phong Nha - Ke Bang National Park, Quang Binh Province, central Vietnam (Nguyen et al. 2009). This is the first record of the species from Laos.

#### Notes

The Laotian specimens differ from the original description of Ziegler et al. (2002) by having somewhat higher femoral and precloacal pore counts in males (36-44 versus 32-42).

### Cyrtodactylus pseudoquadrivirgatus

Rösler, Nguyen, Vu, Ngo & Ziegler, 2008

#### Materials

- **Type status:** Other material. **Occurrence:** recordedBy: T. Calame; individualCount: 2; sex: 2 females; **Location:** country: Laos; stateProvince: Salavan; verbatimLocality: Xe Sap National Protected Area; verbatimElevation: 960 m; verbatimLatitude: 16°09.400’N; verbatimLongitude: 106°49.567’E; **Event:** eventDate: 2012-05-20; **Record Level:** institutionCode: IEBR, NUOL

#### Description

(Fig. 4)

**Specimens examined (n = 2)**. Two adult females (IEBR A.2013.91 & NUOL R-2013.4), collected by T. Calame on 20 May 2012 from Xe Sap NPA, Salavan Province (16°09.400’N, 106°49.567’E, elevation ca. 960 m a.s.l.).

**Morphological characters**. SVL 70.7-83.8 mm, tail regenerated (TaL 72.1-72.6 mm); head depressed (HL/HD 1.6), distinguished from neck; loreal region inflated; snout longer than diameter of orbit (SE/OD 1.9); snout scales small, homogeneous, granular, larger than those in frontal and parietal regions; rostral wider than high with a median suture; supranasals separated from each other posteriorly by a pentagonal internasal; rostral bordered by first supralabial and nostril on each side; nares oval, surrounded by supranasal, rostral, first supralabial, and three enlarged postnasals; eyelid fringe with tiny spines posteriorly; ear oval–shaped, somewhat angular; mental triangular, slightly wider than rostral; postmentals in one pair, enlarged, in broad contact posteriorly, bordered by mental anteriorly, first two infralabials laterally, and one pair of distinctly enlarged gular scales posteriorly, which is separated from each other by two small gular scales; supralabials 8-10; infralabials 7-10; dorsal scales granular to flattened; dorsal tubercles triangular, conical, present on occiput, back and tail base, each surrounded by 10-11 granular scales, in 17-18 irregular longitudinal rows at midbody; ventral scales smooth, medial scales 2–3 times larger than dorsal scales, round, subimbricate, in 39-40 longitudinal rows at midbody; ventrolateral folds with interspersed tubercles; gular region with homogeneous smooth scales; precloacal groove absent; enlarged femoral scales and femoral pores absent; precloacal pores 7-9; postcloacal tubercles 2-3; subcaudals slightly enlarged; dorsal surface of fore and hind limbs with tubercles; fingers and toes without distinct webbing; lamellae under fourth finger 16-19, under fourth toe 17-20. Coloration in preservative: Ground coloration of dorsal head and back blackish brown; a narrow curved black stripe from posterior corner of eye, running above tympanum to the neck, interrupted posteriorly; shoulders, dorsal body blotched, irregular from oval to elongate, dark brown; fore and hind limbs with dark bars; dorsal tail grey with dark brown bands; chin, throat, chest and belly brown; ventral tail marked with light and dark bands; upper and lower lips dark brown (determination after Rösler et al. 2008).

#### Ecology

Specimens were found between 19:40 and 20:10 on a small bush stem ca. 40 cm above the ground, approximately 3 m away from a rocky stream. The surrounding habitat was hill evergreen forest at an elevation of 960 m a.s.l. Within the hill evergreen forest in western Xe Sap NPA the canopy is characterized, in many areas, by the conspicuous presence of emergents of the restricted range conifer *Pinus dalatensis*.

#### Distribution

This species was previously known in Central Vietnam from Quang Tri province southwards to Kon Tum Province (Rösler et al. 2008). Therefore, our record of *Cyrtodactylus pseudoquadrivirgatus* from Salavan Province is the first country record for Laos.

### Lycodon futsingensis

(Pope, 1928)

#### Materials

- **Type status:** Other material. **Occurrence:** recordedBy: V. Q. Luu; individualCount: 1; sex: female; **Location:** country: Laos; stateProvince: Khammouane; verbatimLocality: Hin Nam No National Protected Area; verbatimElevation: 581 m; verbatimLatitude: 17°17.499’N; verbatimLongitude: 106°10.606’E; **Event:** eventDate: 2013-05-14; **Record Level:** institutionCode: VFU

#### Description

(Fig. 5)

**Specimen examined (n = 1)**. VFU A.2013.4, adult female, collected by V. Q. Luu on 14 May 2013 from Noong Ma Commune, Boualapha District, Khammouane Province (17°17.499’N, 106°10.606’E, elevation 581 m a.s.l.), within Hin Nam No NPA.

**Morphological characters.** Total length (TL) 760 mm (SVL 603mm, TaL 157 mm); body subcylindrical; head moderately distinguished from neck, rather flattenned; snout elongate, projecting anteriorly beyond lower jaw; pupil vertically elliptic; maxillary teeth 12/12; rostral distinctly broader than high, partly visible from above; internasals as wide as long, not in contact with loreal; prefrontal less than half length of frontal; frontal hexagonal; parietals longer than wide; nasal paired; loreal 1/1, not in contact with orbit; supralabials 8/8, third to fifth touching the eye, seventh largest; infralabials 9/9, first to fifth bordering chin shields; preocular 1/1; postoculars 2/2, bodering anterior temporals; anterior temporals 2/2, posterior temporals 2/2; dorsal scale rows 17-17-15, smooth; ventrals 209; subcaudals 79, paired; cloacal undivided. Coloration in preservative: Dorsal surface greyish-black with 19-21 grey rings on body and 9 cross-bands on tail; belly cream, anterior part uniform, speckled posteriorly, under tail dark (determination after Vogel et al. 2009, Vogel and David 2010).

#### Ecology

The adult female was collected at ca. 21:30 while moving on the forest floor, near a slow running stream. The surrounding habitat was karst forest at elevation of 581 m a.s.l.

#### Distribution

*Lycodon futsingensis* has been reported from southern China and northern Vietnam (Vogel et al. 2009). This is a new record of the species from Laos and it is approximately about 20 km far from the nearest record of this species in Phong Nha - Ke Bang National Park, Quang Binh Province, Vietnam.

### Lycodon ruhstrati abditus

Vogel, David, Pauwels, Sumontha, Norval, Hendrix, Vu & Ziegler, 2009

#### Materials

- **Type status:** Other material. **Occurrence:** recordedBy: V. Q. Luu; individualCount: 1; sex: male; **Location:** country: Laos; stateProvince: Khammouane; verbatimLocality: Hin Nam No National Protected Area; verbatimElevation: 556 m; verbatimLatitude: 17°17.648’N; verbatimLongitude: 106°10.053’E; **Event:** eventDate: 2013-05-14; **Record Level:** institutionCode: VFU

#### Description

(Fig. 6)

**Specimen examined (n = 1)**. VFU A.2013.5, adult male, collected by V. Q. Luu on 14 May 2013 from Pa Rang region, Noong Ma Commune, Boualapha District, Khammouane Province (17°17.648’N, 106°10.053’E, elevation 556 m a.s.l.), within Hin Nam No NPA.

**Morphological characters.** Total length (TL) 665 mm (SVL 520 mm, TaL 145 mm); body elongate; head moderately distinct from neck, rather flattened; snout projecting anteriorly beyond lower jaw; pupil vertically oval; tail tapered and thin; maxillary teeth 12/12; snout scale broad; rostral distinctly broader than high, partly visible from above; internasals large, pentagonal, not in contact with loreal; prefrontal more than half length of internasal, subrectangular, wider than long, not entering orbit; frontal hexagonal, narrowed posteriorly; parietals longer than wide; nasal paired; loreal 1/1, small, pentagonal, not bordering the eye; supralabials 8/8, third to fifth in contact with the eye, sixth largest; infralabials 10/10, first to fifth bordering chin shields; preocular 1/1; postoculars 2/2, bordering anterior temporal; anterior temporals 2/2; posterior temporals 3/3; dorsal scale rows 17-17-15; five middorsal scales keeled, the outer rows usually smooth; ventrals 224; subcaudals 96, paired; cloacal single. Coloration in preservative: Dorsal surface greyish or blackish, with white and cream cross-bars, 17 on the body, increasing the size at the bottom of each light cross-bands, best marked anteriorly, and becoming dim posteriorly; belly cream, progressively but not extensively speckled with dark grey on the posterior edges of the ventral scales; upper tail as the posterior body, tail rings cream and extending towards the under part of the tail (determination after Vogel et al. 2009, Ziegler et al. 2007).

#### Ecology

The specimen of *Lycodon ruhstrati abditus* was found at 11:00 while moving through a forest path. The surrounding habitat was karst forest at the elevation of 556 m a.s.l.

#### Distribution

*Lycodon ruhstrati* has been known from Taiwan, China and northern Vietnam: *Lycodon ruhstrati ruhstrati* is endemic to Taiwan and the range of *Lycodon ruhstrati abditus* is widespread in the mainland of China and Vietnam (see Vogel et al. 2009). Our finding represents the first record of the species as well as the subspecies, *Lycodon ruhstrati abditus*, from Laos and it is approximately about 20 km far from the type locality of the subspecies in Phong Nha - Ke Bang National Park, Quang Binh Province, Vietnam.

#### Notes

The specimen from Laos differs from the description of Vogel et al. (2009) in having fewer cross-bands on the body (17 versus 19-43).

## Discussion

The recent discoveries of *Cyrtodactylus phongnhakebangensis* and *Cyrtodactylus pseudoquadrivirgatus* in Laos bring the species number of the genus *Cyrtodactylus* known from that country to ten. This is still a low number compared with the *Cyrtodactylus* diversity from neighbouring Vietnam (which currently comprises 29 species, see Ziegler et al. 2013). In central Vietnam’s Phong Nha – Ke Bang National Park two other bent-toed geckos, *Cyrtodactylus cryptus* Heidrich, Rösler, Vu, Böhme & Ziegler, 2007 (Heidrich et al. 2007) and *Cyrtodactylus roesleri* Ziegler, Nazarov, Orlov, Nguyen, Vu, Dang, Dinh & Schmitz, 2010 (Ziegler et al. 2010), are known to occur sympatrically with *Cyrtodactylus phongnhakebangensis* (Loos et al. 2012). In Hin Nam No NPA, our observation also supports the finding of Loos et al. (2012) about niche segregation of *Cyrtodactylus* species. Both *Cyrtodactylus roesleri* and *Cyrtodactylus phongnhakebangensis* were found on karst cliffs in the same habitat (Fig. 7). In Laos, *Cyrtodactylus roesleri* was previously recorded from Phou Hin Boun in Khammouane Province by Teynié and David (2010). This is the first record of the species from Hin Nam No NPA. The new records of *Lycodon futsingensis*, and *Lycodon ruhstrati abditus* from Khammouane Province increase the species number of snakes in Laos to 107. However, the diversity of reptiles in Laos is still poorly studied, particular in Hin Nam No NPA. In the bordering Phong Nha – Ke Bang National Park in Vietnam, Luu et al. (2013) recently provided a list of 101 reptile species including 15 new species and one new subspecies that have been described only from this site since 2000. Therefore, further field research is required to explore the actual herpetofaunal diversity of the largest karst formation in central Laos.

## Supplementary Material

XML Treatment for Cyrtodactylus phongnhakebangensis

XML Treatment for Cyrtodactylus pseudoquadrivirgatus

XML Treatment for Lycodon futsingensis

XML Treatment for Lycodon ruhstrati abditus

## Figures and Tables

**Figure 2a.**
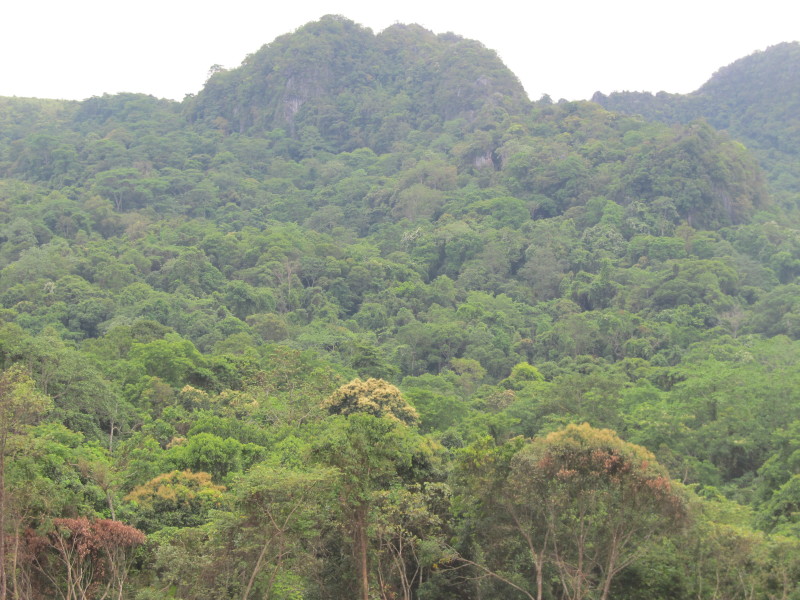
Forest in Hin Nam No NPA

**Figure 2b.**
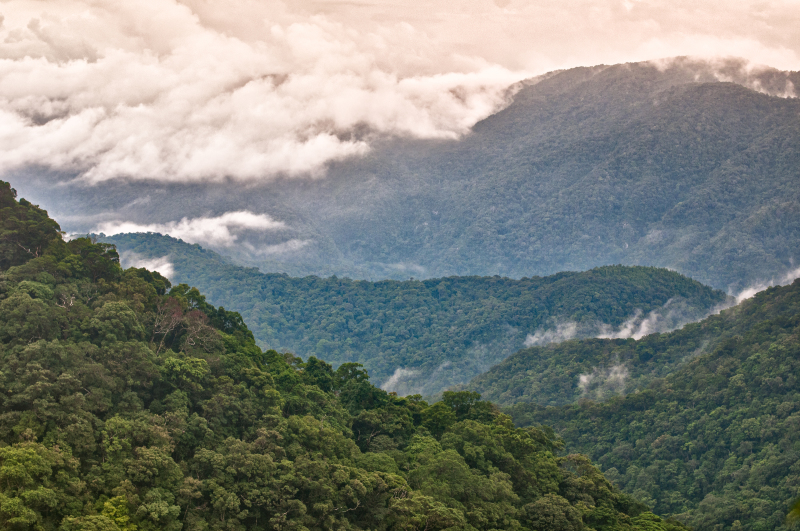
Forest in Xe Sap NPA

**Figure 3a.**
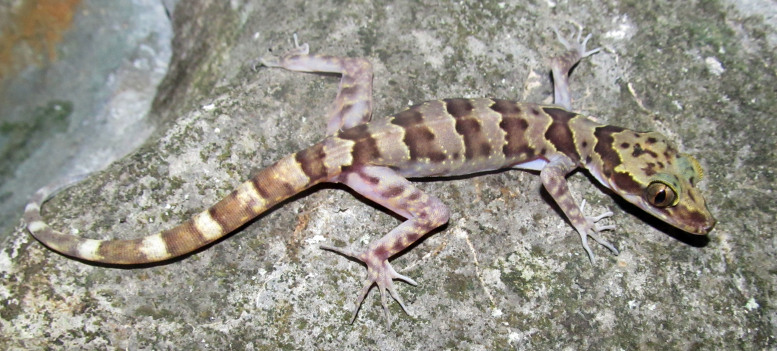
Female

**Figure 3b.**
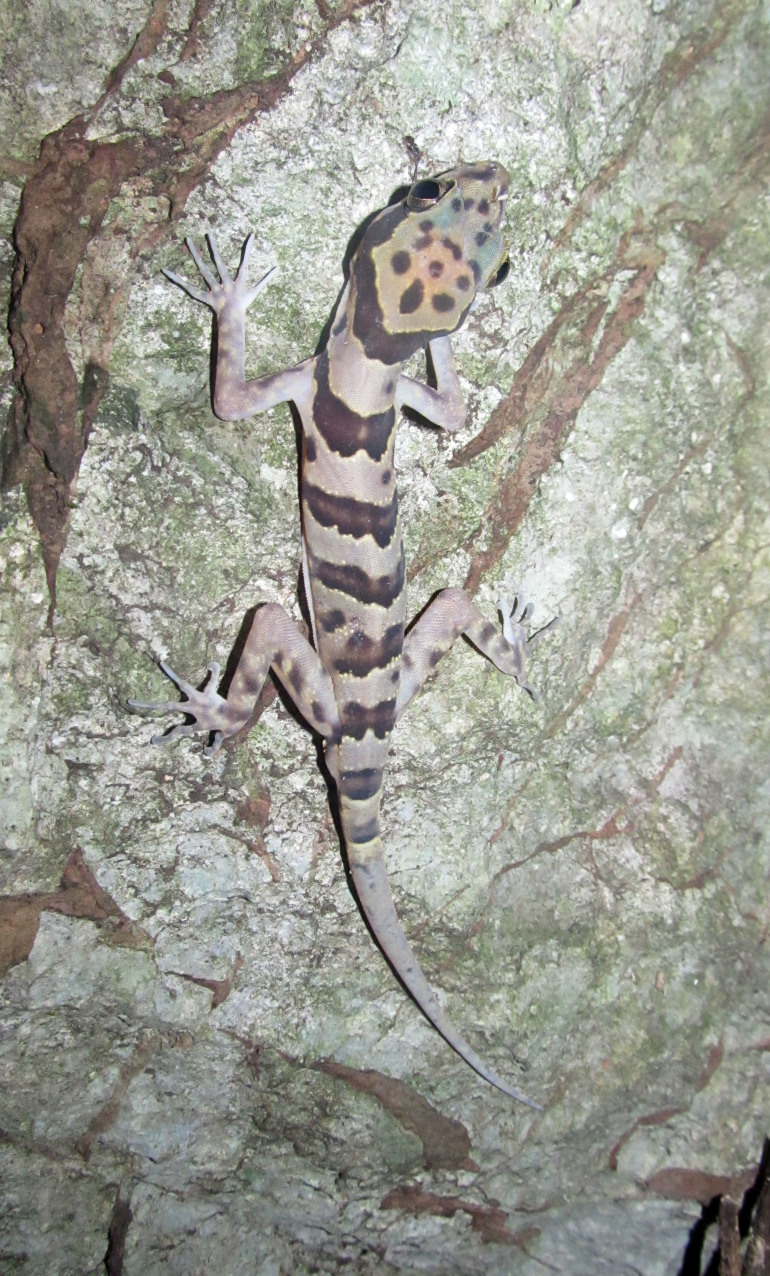
Male

**Figure 4.**
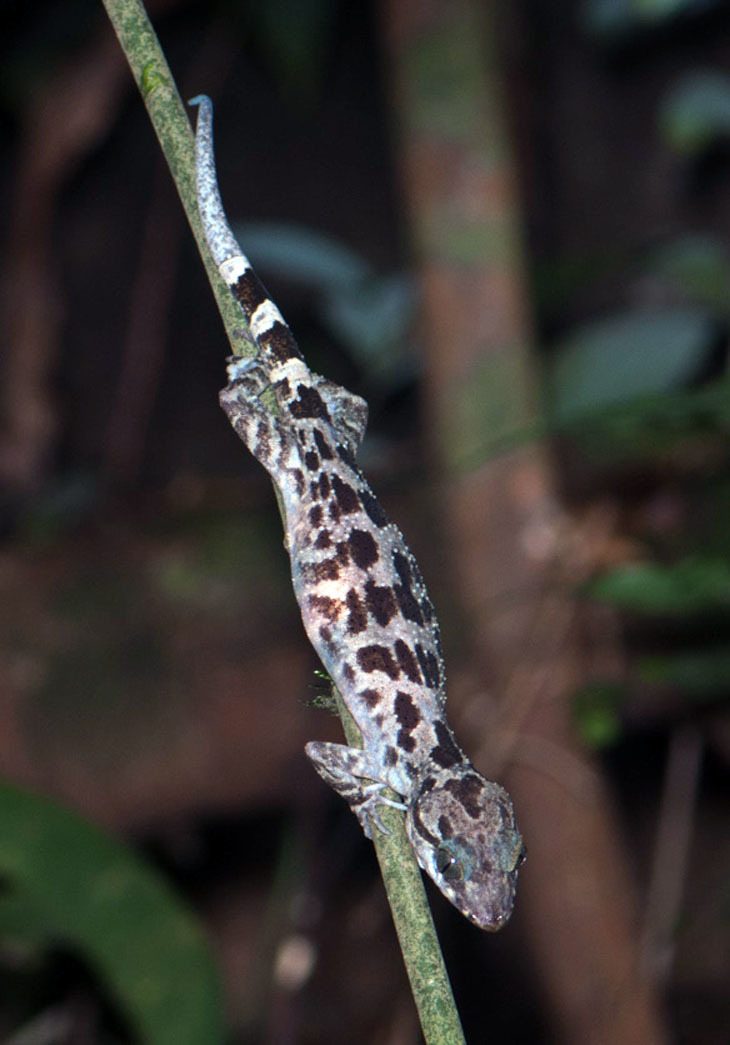
*Cyrtodactylus pseudoquadrivirgatus* from Xe Sap National Protected Area, Salavan Province, Laos. Photo: T. Calame.

**Figure 5a.**
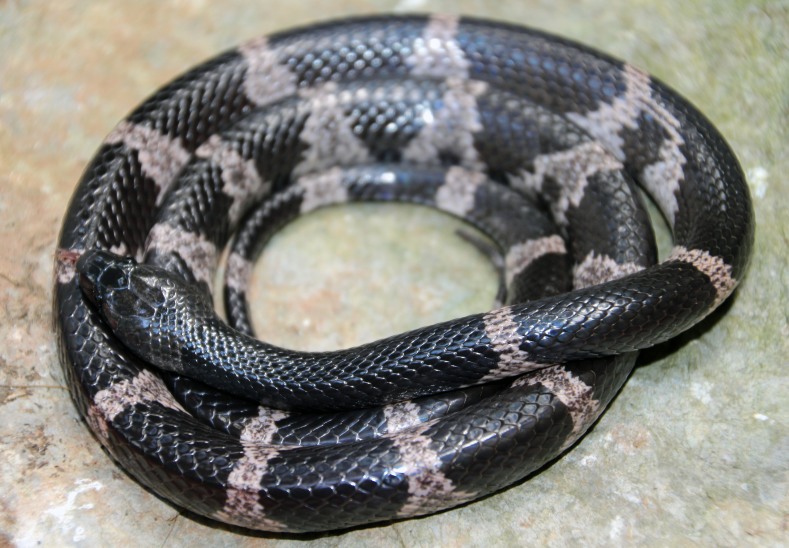
Dorsal view

**Figure 5b.**
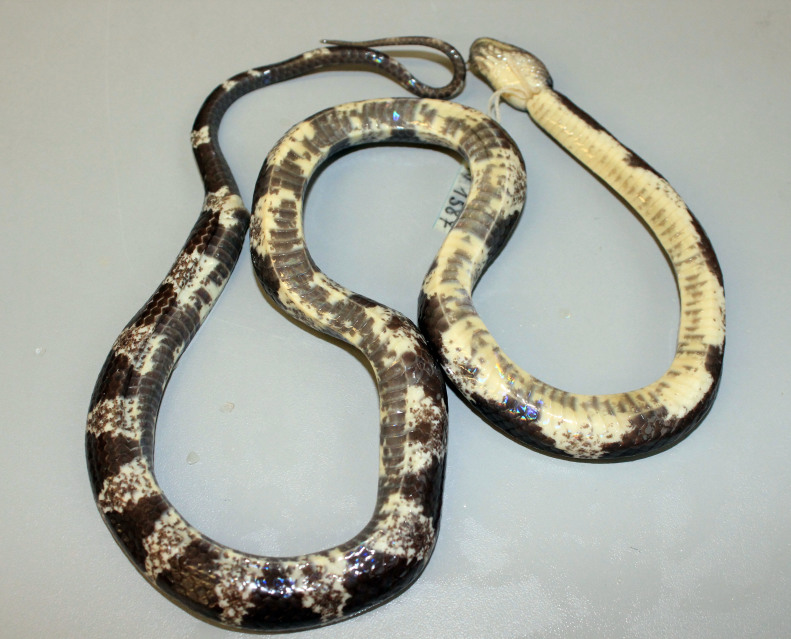
Ventral view

**Figure 6a.**
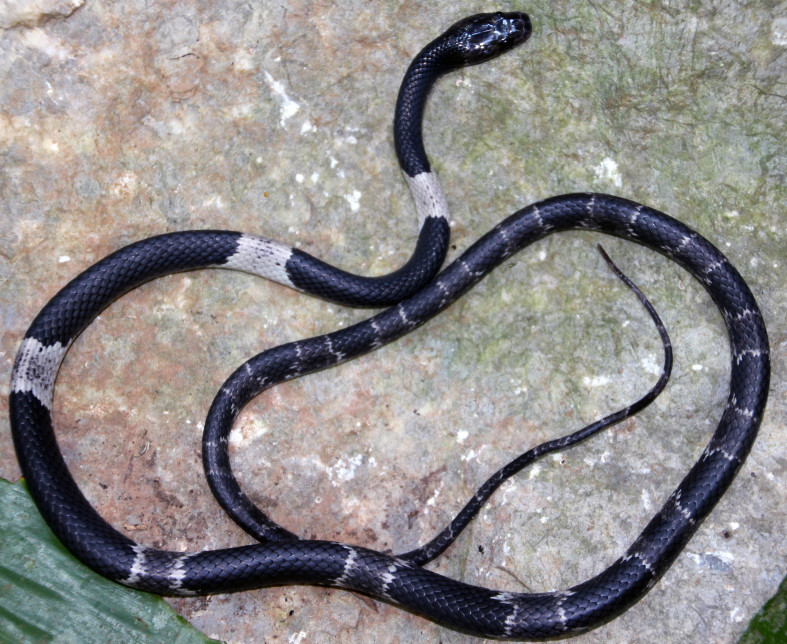
Dorsal view

**Figure 6b.**
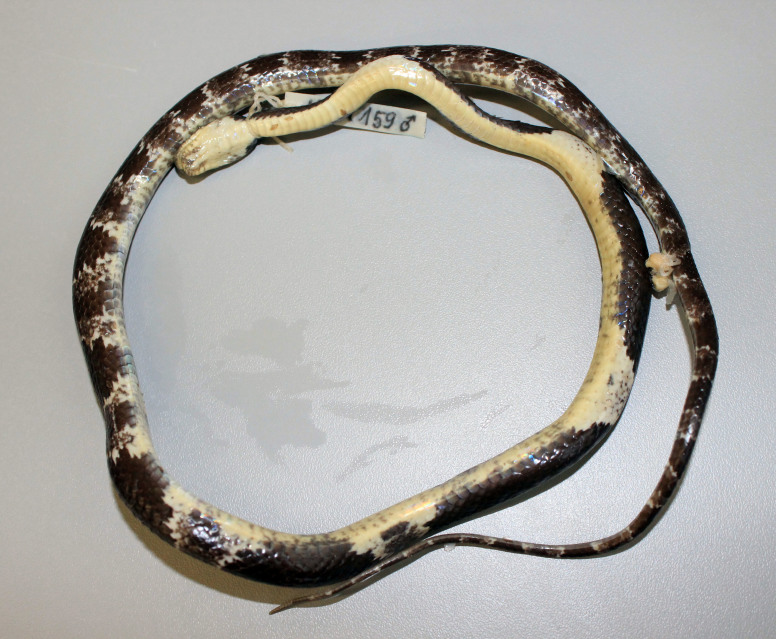
Ventral view

**Figure 7a.**
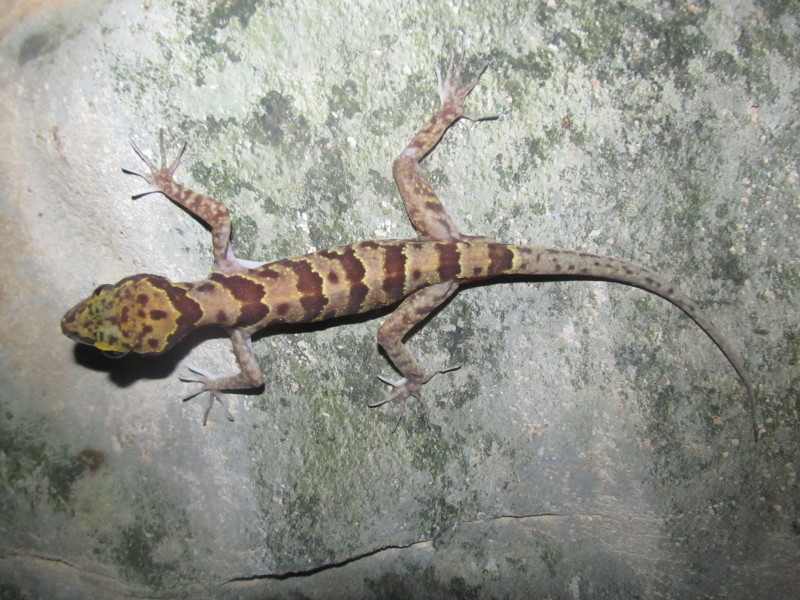
*Cyrtodactylus phongnhakebangensis*

**Figure 7b.**
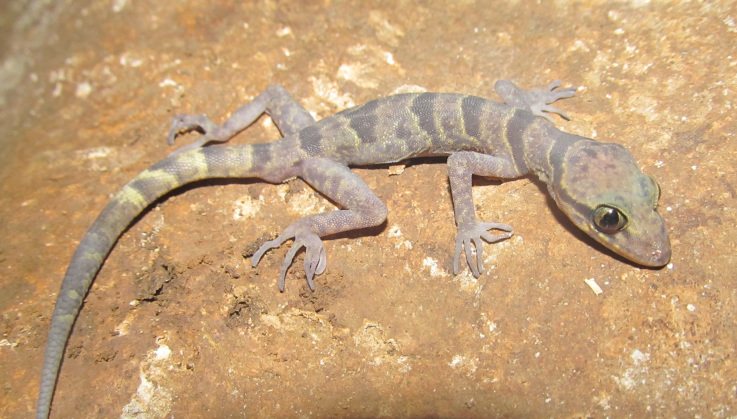
*Cyrtodactylus roesleri*
